# Transcriptome sequencing and network pharmacology-based approach to reveal the effect and mechanism of Ji Chuan Jian against Parkinson’s disease

**DOI:** 10.1186/s12906-023-03999-6

**Published:** 2023-06-03

**Authors:** Yao Wu, Yu Bai, Yan Lu, Zhennian Zhang, Yang Zhao, Sirui Huang, Lili Tang, Yan Liang, Yue Hu, Chengcheng Xu

**Affiliations:** 1 Department of Neurology, Jiangsu Province Hospital of Chinese Medicine, Affiliated Hospital of Nanjing University of Chinese Medicine, Nanjing, Jiangsu China; 2 School of Chinese Medicine, School of Integrated Chinese and Western Medicine, Nanjing University of Chinese Medicine, Nanjing, Jiangsu China; 3 Nanjing Hospital of Chinese Medicine Affiliated to Nanjing University of Chinese Medicine, Nanjing, Jiangsu China

**Keywords:** Transcriptome sequencing, Ji Chuan Jian, Network pharmacology, Parkinson’s disease, Mechanism

## Abstract

**Background:**

*Ji Chuan Jian* (JCJ), a classic Traditional Chinese Medicine (TCM) formula, has been widely applied in treating Parkinson’s disease (PD) in China, However, the interaction of bioactive compounds from JCJ with the targets involved in PD remains elusive.

**Methods:**

Based on the transcriptome sequencing and network pharmacology approaches, the chemical compounds of JCJ and gene targets for treating PD were identified. Then, the Protein-protein interaction (PPI) and “Compound-Disease-Target” (C-D-T) network were constructed by using of Cytoscape. Gene Ontology (GO) enrichment analysis and Kyoto Encyclopedia of Genes and Genomes (KEGG) pathway enrichment analysis were applied to these target proteins. Finally, AutoDock Vina was used for applying molecular docking.

**Results:**

In the present study, a total number of 2669 differentially expressed genes (DEGs) were identified between PD and healthy controls using whole transcriptome RNA sequencing. Then, 260 targets of 38 bioactive compounds in JCJ were identified. Of these targets, 47 were considered PD-related targets. Based on the PPI degree, the top 10 targets were identified. In C-D-T network analysis, the most important anti-PD bioactive compounds in JCJ were determined. Molecular docking revealed that potential PD-related targets, matrix metalloproteinases-9 (MMP9) were more stably bound with naringenin, quercetin, baicalein, kaempferol and wogonin.

**Conclusion:**

Our study preliminarily investigated the bioactive compounds, key targets, and potential molecular mechanism of JCJ against PD. It also provided a promising approach for identifying the bioactive compounds in TCM as well as a scientific basis for further elucidating the mechanism of TCM formulae in treating diseases.

**Supplementary Information:**

The online version contains supplementary material available at 10.1186/s12906-023-03999-6.

## Introduction

Parkinson’s disease (PD) is a multifactorial neurodegenerative movement disorder that is associated with the progressive impairment of voluntary motor control [1]. The clinical symptoms of PD mainly include motor symptoms such as bradykinesia, static tremor, postural instability, and hypokinesia [2], the non-motor symptoms such as autonomic nerve dysfunction, gastrointestinal dysfunction, bladder dysfunction, and even fatigue are also considered as important components of PD [3]. The major pathological changes of PD are the loss of dopaminergic neurons in the substantia nigra of the midbrain leading to the significant reduction of dopamine content in the striatum, and the presence of Lewy body (LB) in the substantia nigra and locus coeruleus [4, 5]. In recent years, the incidence rate of PD is increasing rapidly. It is estimated that by 2030, PD patients in China will reach 4.94 million, accounting for about 50% of patients in the world [6]. PD treatment involves pharmacologic approaches (mainly levodopa) and nonpharmacologic approaches (i.g., exercise or physical therapies) [7]. However, to date, for all patients with PD, treatment is symptomatic, no agents have been shown to have unequivocal evidence of disease-modifying effects in PD [8]. Thus, a better understanding of PD’s pathogenesis, alongside the exploration of novel medicine approaches is urgently needed.

*Ji Chuan Jian* (JCJ) is a classic Traditional Chinese Medicine (TCM) formula first mentioned in a famous ancient medicine treatise, “Jing Yue Quan Shu”, which was written by Zhang Jingyue, a physician in the Ming Dynasty. JCJ is composed of six herbal drugs: *Angelica sinensis* (Chinese name: *Danggui* (DG)), *Achyranthes bidentata* (Chinese name: *Niuxi* (NX)), *Aurantii Fructus* (Chinese name: *Zhiqiao* (ZQ)), *Rhizoma Cimicifugae* (Chinese name: *Shengma* (SM)), *Cistanche deserticola* (Chinese name: *Roucongrong* (RCR)), *Alisma orientalis* (Chinese name: *Zexie* (ZX)). In clinical practice, JCJ is used to improve the constipation symptoms of patients with PD, alleviate the motor symptoms and reduce the adverse reactions of anti-PD drugs in a long term [9, 10]. In experimental studies, Achyranthes bidentata polypeptide, a bioactive substance extracted from *Achyranthes bidentata*, showed a dopaminergic neuronal protective effect in PD models [11]. Another agent Echinacoside, the major active constituent of *Cistanche deserticola*, was found to exert neuroprotection in PD through IL-6/JAK2/STAT3 and NLRP3/Caspase-1/IL-1β anti-inflammatory pathway [12, 13]. Although JCJ has been used for a long time and has received significant experimental and clinical support, its active ingredients, putative target genes, and underlying mechanisms for treating PD have yet to be fully elucidated.

**Fig. 1:**
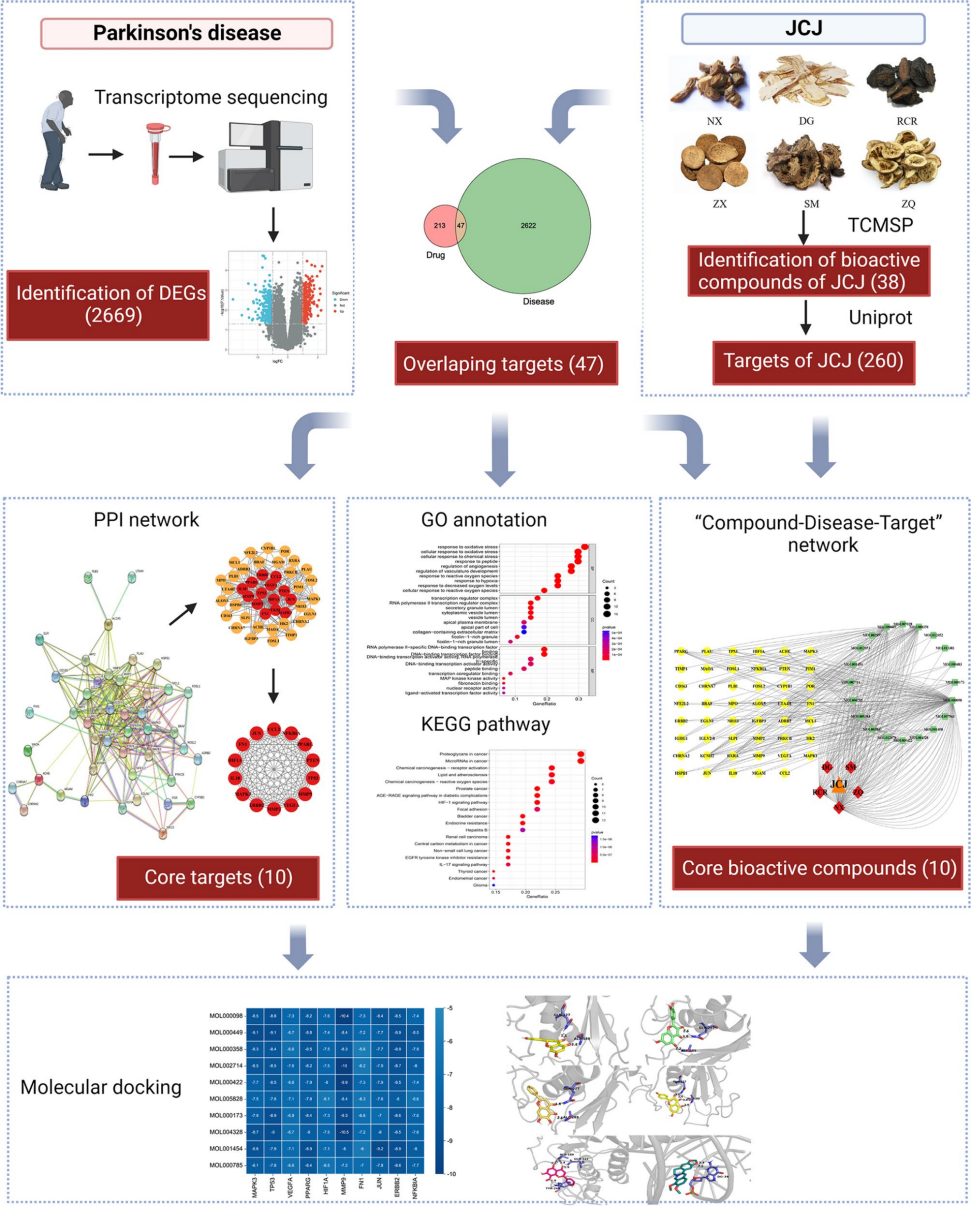
Workflow of the study design. JCJ for the potential treatment of PD based on transcriptome sequencing and network pharmacology-based approach

In the present study, we used transcriptome sequencing and a network pharmacology-based approach, as well as molecular docking technology to explore the relevant biological pathways associated with JCJ in treating PD. This is the first systematic research on the effects and mechanisms of JCJ, which will provide a theoretical basis for clinical application. A workflow chart is shown in Fig. 1.

## Materials and methods

### Peripheral blood samples

Blood samples obtained from PD patients (n = 12) or healthy controls (n = 12) with written informed consent were selected from the Nanjing Hospital of Chinese Medicine Affiliated to Nanjing University of Chinese Medicine from July 2019 to March 2021. All procedures performed in this study involving patients were in accordance with the 1964 Helsinki Declaration and were approved by the Ethics Committee of Nanjing Hospital of Chinese Medicine Affiliated to Nanjing University of Chinese Medicine Hospital (No. KY2022376). Informed consent was obtained from all individual participants prior to any study-related procedures. The inclusion and exclusion criteria were as follows. Inclusion criteria: Inclusion criteria: (1) the diagnosis of PD by a neurologist specialized in movement disorders, according to the clinical criteria proposed by Gelb; (2) stable medication usage; (3) age equal or greater than 60 years. (4) Hoehn and Yahr scale stage 1–3. (5) Mini-Mental State Examination score less than 23 during the initial assessment. Exclusion criteria: (1) a history of any significant neurological disorder; (2) suffered from unstable cardiovascular disease; (3) suffered from other uncontrolled conditions that would interfere with the participant’s safety. Healthy controls were selected from the hospital health examination center and were matched for age (within ± 5 years).

### RNA extraction and whole transcriptome RNA sequencing

A total volume of 2ml of blood obtained from all individual participants was extracted using BD PAXgene blood RNA tubes. Then, the blood samples were incubated with PAXgene tubes until the blood cells were completely dissolved. The total RNA was extracted using the TRIzol reagent (Leagene Biotechnology, Beijing, China) following the manufacturer’s protocol. Afterward, isolated RNA was treated with DNAse I and silica-membrane purification (RNeasy kit, Qiagen, Hilden, Germany) and further for Illumina sequencing. An equivalent of 50ng RNA was utilized for the RNA sample preparations. Single reads of a length of 50 base pairs (bp) were sequenced on an Illumina HiSeq2500 according to the manufacturer’s protocol. RNAseq reads were provided in compressed Sanger FASTQ format. Gene expression was calculated on the gene level as raw counts and transcripts per million (TPM).

### Identification of differentially expressed genes (DEGs) between PD and healthy controls

Based on the data from whole transcriptome RNA sequencing, gene expression of 24 samples, including 12 blood samples of PD and 12 blood samples of controls were analyzed using Illumina Sequencing Analysis Viewer (Illumina, San Diego, USA). The DEGs were first screened with the restriction of |log2 (Fold-Change)| > 0.5 and p < 0.05 [14–16].

### Data source of network pharmacology

All ingredients of JCJ were collected from the Traditional Chinese Medicine System Pharmacology (TCMSP) Database [17] (https://tcmspw.com/tcmsp.php). In the TCMSP system, Oral bioavailability (OB) and Drug likeness (DL) is the major parameter to evaluate the quality of active ingredients. As recommended in several studies, the ingredients with OB ≥ 30% and DL ≥ 0.18 are considered to have better pharmacologic effects and can be selected as candidate ingredients for the next step [18]. All the target proteins corresponding to each molecular were also collected from TCMSP and transformed into gene symbols using the UniProt knowledge database (https://www.uniprot.org/).

### Protein-protein interaction (PPI) network construction

Firstly, common targets of PD-related DEGs and JCJ were analyzed using the Venn package in R. To further study the interactions between JCJ and PD-related targets, STRING (Search Tool for the Retrieval of Interacting Genes/Proteins https://string-db.org/) was used for predicting protein-protein interactions [19]. The common target proteins with species were set as “Homo sapiens” and the confidence score was set larger than 0.4. Subsequently, the PPI results were exported from STRING and imported into Cytoscape 3.8.0 software [20] to realize visualization and screen out the core targets. The protein ID were obtained from Uniprot database (https://www.uniprot.org/).

### Constructing the “Compound-Disease-Target” network

To better demonstrate the mechanism of action of JCJ in PD treatment, we constructed the “Compound-Disease-Target” (C-D-T) network. The networks were built using the Cytoscape 3.8.0 software. In the network plot, nodes represent the PD/JCJ/ingredients/target genes, while edges stand for that they are linked with each other [21, 22]. This “C-D-T” network facilitates scientific interpretation of the complicated relationships among compounds, genes, targets, and diseases.

### Functional enrichment analysis

The biological function and pathway of the core targets were analyzed by Gene Ontology (GO) and Kyoto Encyclopedia of Genes and Genomes (KEGG) enrichment. GO enrichment analysis interprets the biological process (BP), cellular component (CC), and molecular function (MF) of target genes in terms of gene function. In our study, we used clusterProfiler package in R to perform the analysis, parameters were set as follows: pvalueCutoff = 0.05, qvalueCutoff = 0.2 [23]. For KEGG pathway analysis, overlapping target genes were executed by the R Project. Only functional terms and pathways with p values < 0.05 were considered statistically significant.

### Molecular docking validation

The top 10 target proteins in the PPI network were selected for molecular docking. The 3D structure of the targets was downloaded in the Protein Data Bank (PDB) database (http://www.rcsb.org/). The 2D structure of the drug small molecule was downloaded from the TCMSP Database. The target protein was hydrogenated and converted to * pdbqt format by AutoDock software. Subsequently, the * pdbqt format protein receptor files and ligands were imported to AutoDock tools to construct mating pockets of docking. The docking process was provided with AutoDock tools 1.5.6 with Vina and visualized using the PyMol software (version 2.3.0) [24].

## Results

### Identification of DEGs between PD and healthy controls based on transcriptome sequencing

**Fig. 2:**
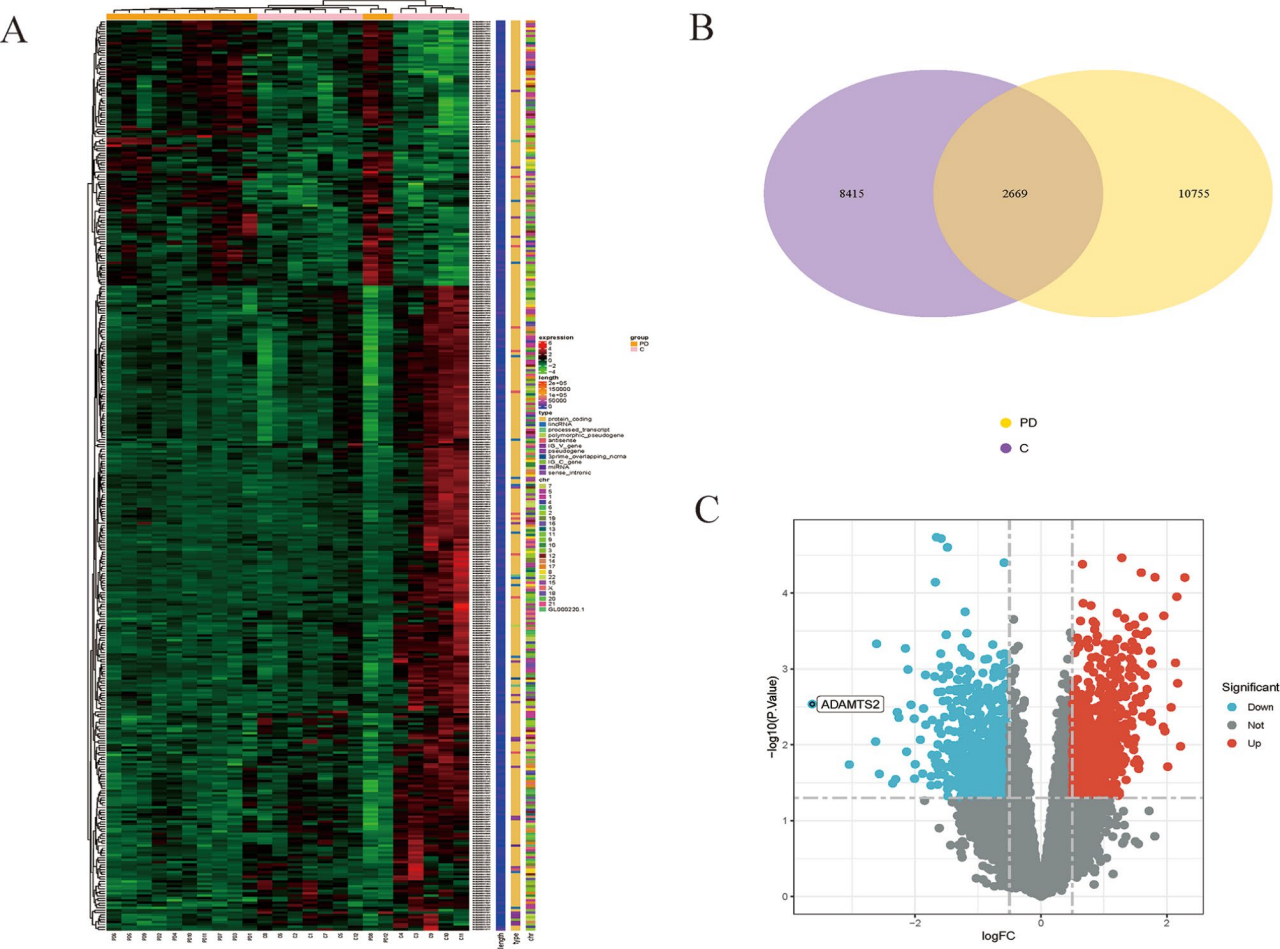
Identification of DEGs between PD and controls using transcriptome sequencing. **(A)** Heatmaps of all DEGs between PD samples and controls. Red and green in heatmaps indicated unigenes with higher and lower expression levels. **(B)** Venn diagram showing the number of identified genes in PD and healthy controls. **(C)** Volcano plot of DGEs, Y-axis shows the value of -log10 (FDR), x-axis shows the log2 (fold change) between PD samples and controls. Red and blue spots represented up-regulated and down-regulated DEGs, while grey spots indicate genes with no significant change between two groups

Transcriptomic data were obtained from six different cDNA libraries. After removing the adaptor sequence and the low-quality sequence, there were more than 40 million clean reads were obtained from each transcriptome, with Q20 and Q30 were more than 97% and 93%, respectively (Supplemental Table 1). Among clean reads, 96.92–97.83% from each transcriptome were mapped, of which 86.82–97.23% mapped to exons, 1.72–10.14% mapped to introns, and 0.99–3.04% mapped to intergenic regions (Supplemental Tables 2 and 3). Based on the data of whole transcriptome RNA sequencing, the heatmap of DEGs was roughly shown in Fig. 2a, a total number of 2669 DEGs were identified between PD and healthy controls (Fig. 2b and c). Among them, there were 1914 down-regulated genes and 755 up-regulated genes.

### Collection of targets of bioactive compounds in JCJ and PD-related targets

To identify the active chemical compounds from JCJ, we applied TCMSP system. In the system, OB ≥ 30% and DL ≥ 0.18 were set as the criteria for screening candidate compounds from each herbal medicine in JCJ. A total number of 38 initial chemical components of JCJ were obtained from TCMSP database (Table 1). Then, the protein targets of these active compounds in JCJ were identified in Uniprot database for normalization, and a total number of 938 targets were obtained, including 81 targets in *Rhizoma Cimicifugae*, 121 targets in *Aurantii Fructus*, 69 targets in *Angelica sinensis*, 438 targets in *Achyranthes bidentata*, 220 targets in *Cistanche deserticola* and 9 targets in *Alisma orientalis* (Supplemental Table 4). After removing duplicate candidates, 260 targets were finally identified.

**Table 1:** Bioactive compounds of JCJ

Mol ID	Molecule Name	OB(%)	DL	Herb name
MOL000358	beta-sitosterol	36.91	0.75	*Angelica sinensis, Achyranthes bidentata, Aurantii Fructus*
MOL000449	stigmasterol	43.83	0.76	*Angelica sinensis, Achyranthes bidentata, Rhizoma Cimicifugae*
MOL001006	poriferasta-7,22E-dien-3beta-ol	42.98	0.76	*Achyranthes bidentata*
MOL012461	28-norolean-17-en-3-ol	35.93	0.78	*Achyranthes bidentata*
MOL001454	berberine	36.86	0.78	*Achyranthes bidentata*
MOL001458	coptisine	30.67	0.86	*Achyranthes bidentata*
MOL000173	wogonin	30.68	0.23	*Achyranthes bidentata*
MOL002643	delta 7-stigmastenol	37.42	0.75	*Achyranthes bidentata*
MOL002714	baicalein	33.52	0.21	*Achyranthes bidentata*
MOL002776	Baicalin	40.12	0.75	*Achyranthes bidentata, Rhizoma Cimicifugae*
MOL002897	epiberberine	43.09	0.78	*Achyranthes bidentata*
MOL003847	Inophyllum E	38.81	0.85	*Achyranthes bidentata*
MOL000422	kaempferol	41.88	0.24	*Achyranthes bidentata*
MOL004355	Spinasterol	42.98	0.76	*Achyranthes bidentata*
MOL000785	palmatine	64.6	0.65	*Achyranthes bidentata*
MOL000085	beta-daucosterol_qt	36.91	0.75	*Achyranthes bidentata*
MOL000098	quercetin	46.43	0.28	*Achyranthes bidentata, Cistanche deserticola*
MOL005320	arachidonate	45.57	0.2	*Cistanche deserticola*
MOL005384	suchilactone	57.52	0.56	*Cistanche deserticola*
MOL007563	Yangambin	57.53	0.81	*Cistanche deserticola*
MOL008871	Marckine	37.05	0.69	*Cistanche deserticola*
MOL000359	sitosterol	36.91	0.75	*Alisma orientalis, Rhizoma Cimicifugae*
MOL000831	Alisol B monoacetate	35.58	0.81	*Alisma orientalis*
MOL000849	16β-methoxyalisol B monoacetate	32.43	0.77	*Alisma orientalis*
MOL000853	alisol B	36.76	0.82	*Alisma orientalis*
MOL000856	alisol C monoacetate	33.06	0.83	*Alisma orientalis*
MOL002464	1-Monolinolein	37.18	0.3	*Alisma orientalis*
MOL000862	[(1 S,3R)-1-[(2R)-3,3-dimethyloxiran-2-yl]-3-[(5R,8 S,9 S,10 S,11 S,14R)-11-hydroxy-4,4,8,10,14-pentamethyl-3-oxo-1,2,5,6,7,9,11,12,15,16-decahydrocyclopenta[a]phenanthren-17-yl]butyl] acetate	35.58	0.81	*Alisma orientalis*
MOL012052	Tuberosine A	102.67	0.34	*Rhizoma Cimicifugae*
MOL012053	cimicifugic acid	83.02	0.45	*Rhizoma Cimicifugae*
MOL012078	visamminol	50.01	0.23	*Rhizoma Cimicifugae*
MOL012081	(20r,24r)-24,25-epoxy-3-beta-(beta-d-xylopyranosyloxy)-9,19-cyclolanost-7-ene-16,23-dione_qt	40.1	0.76	*Rhizoma Cimicifugae*
MOL001924	paeoniflorin	53.87	0.79	*Rhizoma Cimicifugae*
MOL000483	(Z)-3-(4-hydroxy-3-methoxy-phenyl)-N-[2-(4-hydroxyphenyl)ethyl]acrylamide	118.35	0.26	*Rhizoma Cimicifugae*
MOL013381	Marmin	38.23	0.31	*Aurantii Fructus*
MOL002341	Hesperetin	70.31	0.27	*Aurantii Fructus*
MOL004328	naringenin	59.29	0.21	*Aurantii Fructus*
MOL005828	nobiletin	61.67	0.52	*Aurantii Fructus*

**Fig. 3:**
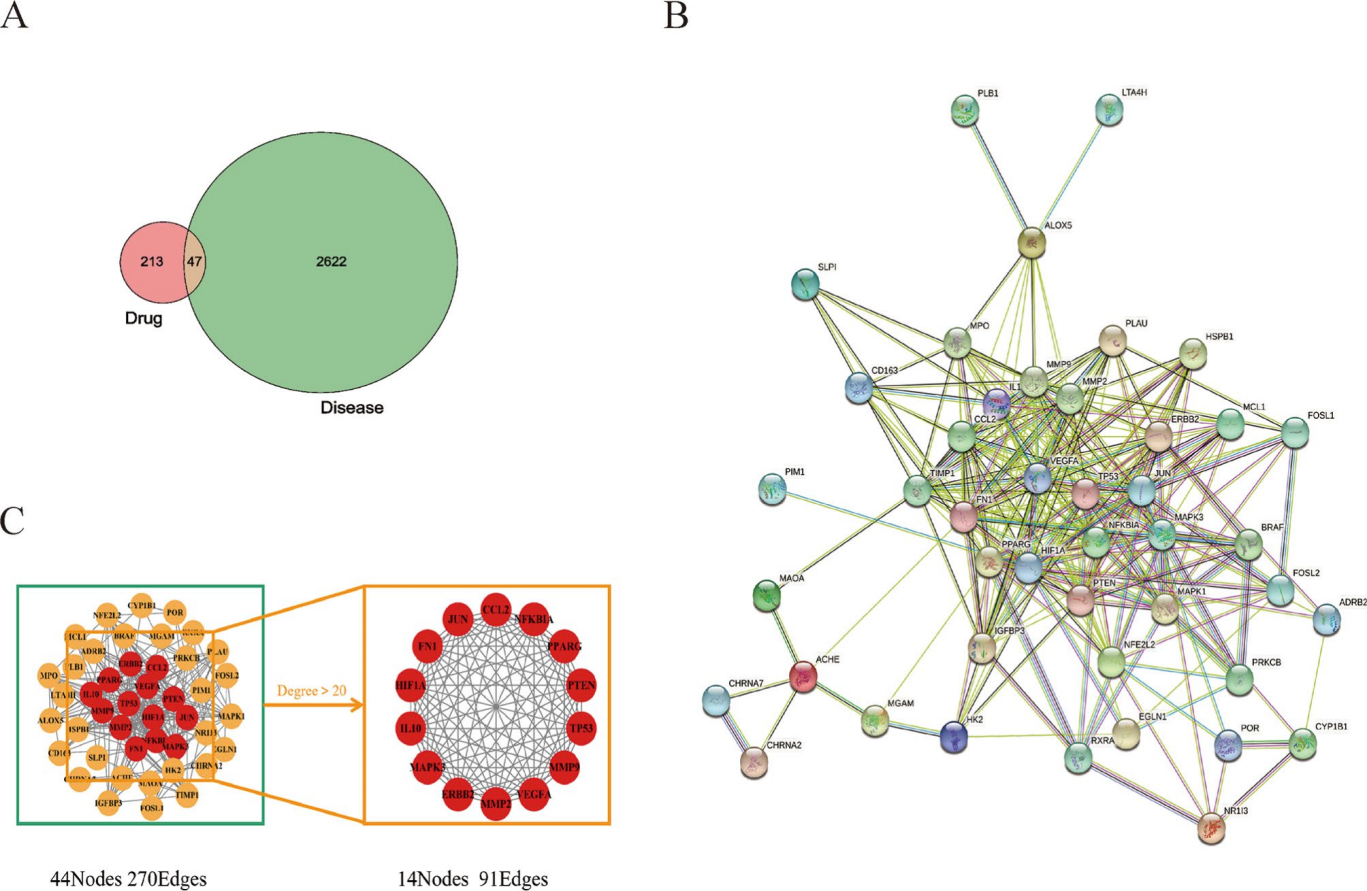
The potential target for JCJ in the treatment of PD. **(A)** Venn diagram showing the potential targets for JCJ in the treatment of PD. **(B)** PPI network of potential PD targets by JCJ. Each node represents relevant targets, and edges stand for protein-protein associations, including known interactions. **(C)** Core PPI network of core proteins, which contains 14 nodes and 9 edges. Red indicates a higher degree, and yellow represents a lower degree

As shown in Fig. 3a, after mapping JCJ-related targets and PD-related targets in a Venn diagram, 47 overlapping targets were accessed. To further explore the mechanism of these targets, the PPI network was built by inputting 47 overlapping targets into the STRING Database (Fig. 3b). There was a total of 44 nodes and 270 Edges. The nodes with Degree > 20 was regarded as the key node in the network. In the core PPI network in Fig. 3c, the target nodes with the highest degree values were MAPK3, TP53, VEGFA, PPARG, HIF1A, MMP9, FN1, JUN, ERBB2, NFKBIA, MMP2, CCL2, IL10, PTEN. Among them, MAPK3, TP53, VEGFA had the highest degree values. The top-10 JCJ treatment related targets were listed in Table 2.

**Table 2:** Top 10 target of JCJ-related targets for PD treatment

Gene ID	Protein ID	Degree	Gene Name
NM_001109891.2	Q16644	28	MAPK3
NM_001276761.3	Q12888	26	TP53
NM_001317010.1	P15692	26	VEGFA
NM_001354666.3	P37231	25	PPARG
NM_001243084.2	Q16665	25	HIF1A
NM_004994.3	P14780	24	MMP9
NM_001306132.2	P02751	23	FN1
NM_002228.4	P05412	23	JUN
NM_001382782.1	P04626	21	ERBB2
NM_020529.3	P25963	21	NFKBIA

### The construction of “C-D-T” network

**Fig. 4:**
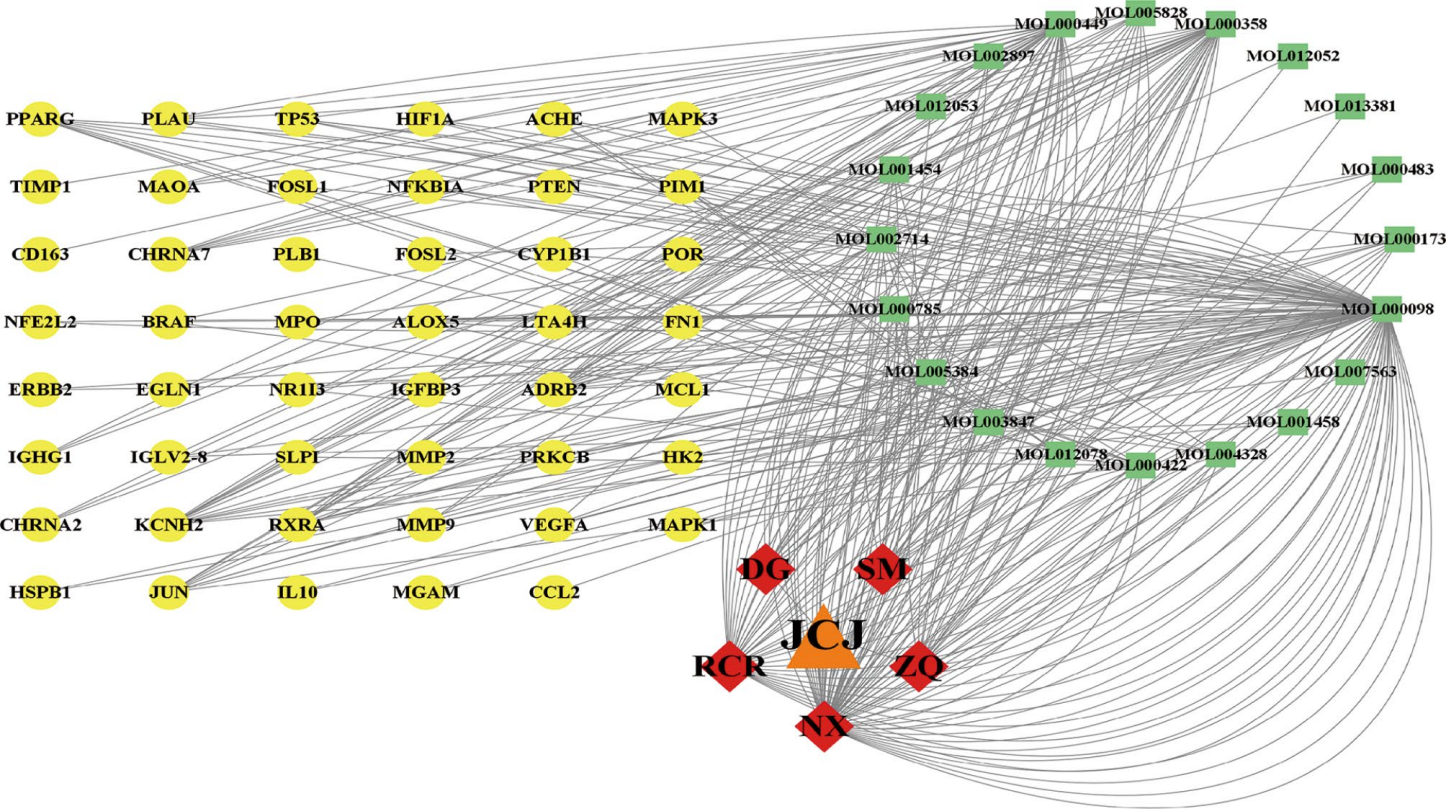
“Compound-Disease-Target” network of treatment with JCJ for PD. Yellow ellipse nodes indicated target genes in PD, green square nodes stand for the bioactive compounds from JCJ, red diamond nodes represent five herbs in JCJ: *Angelica sinensis* (DG), *Rhizoma Cimicifugae* (SM), *Cistanche deserticola* (RCR), *Aurantii Fructus* (ZQ), *Achyranthes bidentata* (NX).

The association between 20 active compounds in JCJ and 47 PD-related target genes was visualized by “C-D-T” network using Cytoscape (Fig. 4). In “C-D-T” network, green square nodes stand for the bioactive compounds from *Angelica sinensis*, *Rhizoma Cimicifugae*, *Cistanche deserticola*, *Aurantii Fructus*, *Achyranthes bidentata* in JCJ. The red diamond nodes represent these five herbs in JCJ. Yellow ellipse nodes indicated target genes in PD. Nodes with a greater number of edges have higher degree values, suggesting greater significance in this network. The top bioactive compounds may be the critical nodes in the network and possess an important anti-PD effect, including quercetin, stigmasterol, beta-sitosterol, baicalein, kaempferol, nobiletin, wogonin, naringenin, berberine, palmatine (Table 3).

**Table 3:** Top 10 core component from JCJ for PD treatment

Mol ID	Degree	Name	Source
MOL000098	112	quercetin	*Achyranthes bidentata, Cistanche deserticola*
MOL000449	40	Stigmasterol	*Angelica sinensis, Achyranthes bidentata*,
MOL000358	36	beta-sitosterol	*Angelica sinensis, Achyranthes bidentata, Cistanche deserticola, Fructus Aurantii*
MOL002714	16	baicalein	*Achyranthes bidentata*
MOL000422	16	kaempferol	*Achyranthes bidentata*
MOL005828	14	nobiletin	*Fructus Aurantii*
MOL000173	10	wogonin	*Achyranthes bidentata*
MOL004328	8	naringenin	*Fructus Aurantii*
MOL001454	8	berberine	*Achyranthes bidentata*
MOL000785	8	palmatine	*Achyranthes bidentata*

### Enrichment analysis

**Fig. 5:**
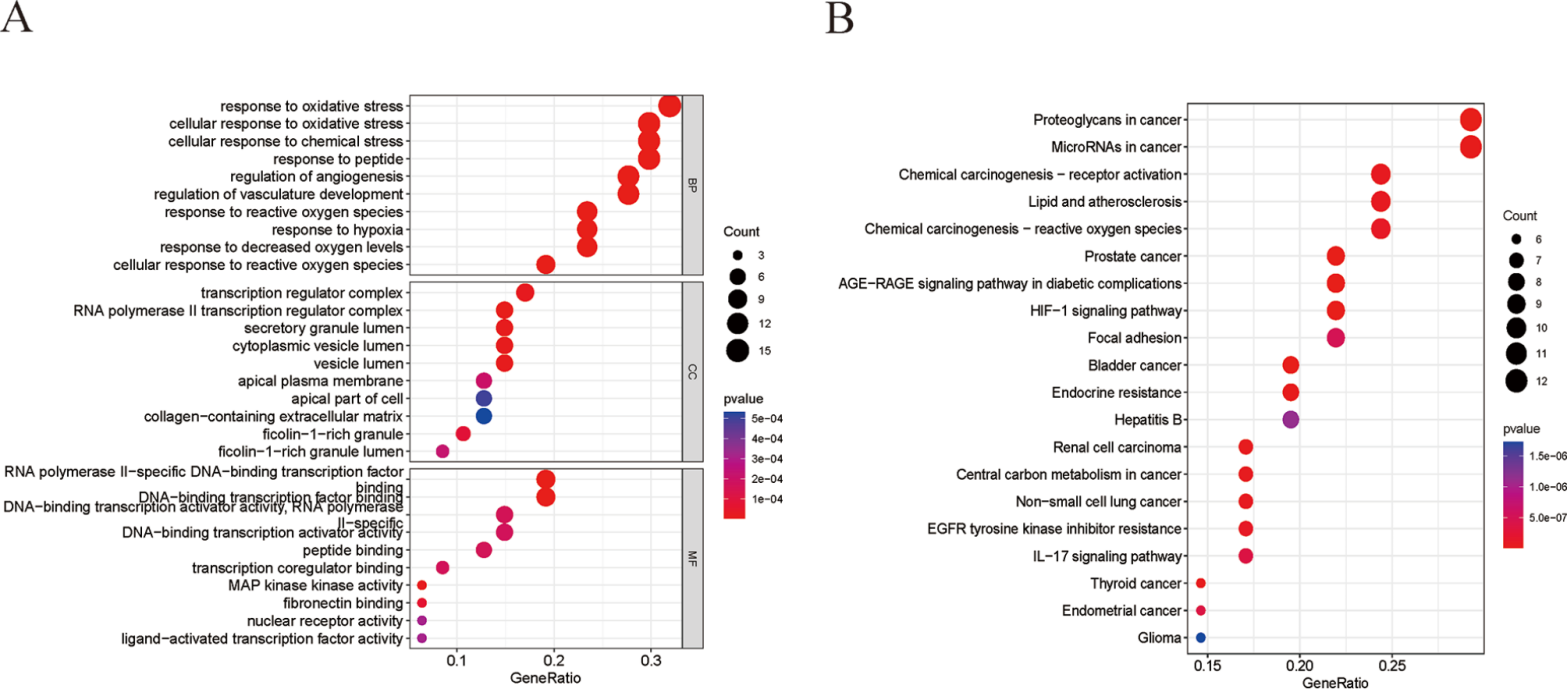
Enrichment analysis of JCJ-PD genes. **(A)** GO enrichment analysis of key targets of JCJ in treatment of PD, the BP, CC, and MF terms were plotted on the y-axis. **(B)** KEGG pathway analysis of key targets of JCJ in treatment of PD. Dot plot showing the top 20 enriched KEGG pathways

To further elucidate the underlying mechanism of the key potential anti-PD genes of JCJ, GO enrichment analysis and KEGG pathway enrichment analysis were carried out on 47 intersection targets. The GO analysis consists of BP, CC, and MF. As shown in Fig. 5a, the key targets of JCJ acting on PD were involved in oxidative stress, chemical stress, regulation of angiogenesis, transcription regulator complex, RNA polymerase II transcription regulator complex, RNA polymerase II-specific DNA binding transcription factor binding, DNA binding transcription factor binding, and MAP kinase kinase activity. For KEGG pathways analysis, there were several pathways related to PD were obtained with the condition of P < 0.05, The most significantly enriched signaling pathways were the HIF-1 signaling pathway and the IL-17 signaling pathway (Fig. 5b).

### Molecular docking analysis

**Fig. 6:**
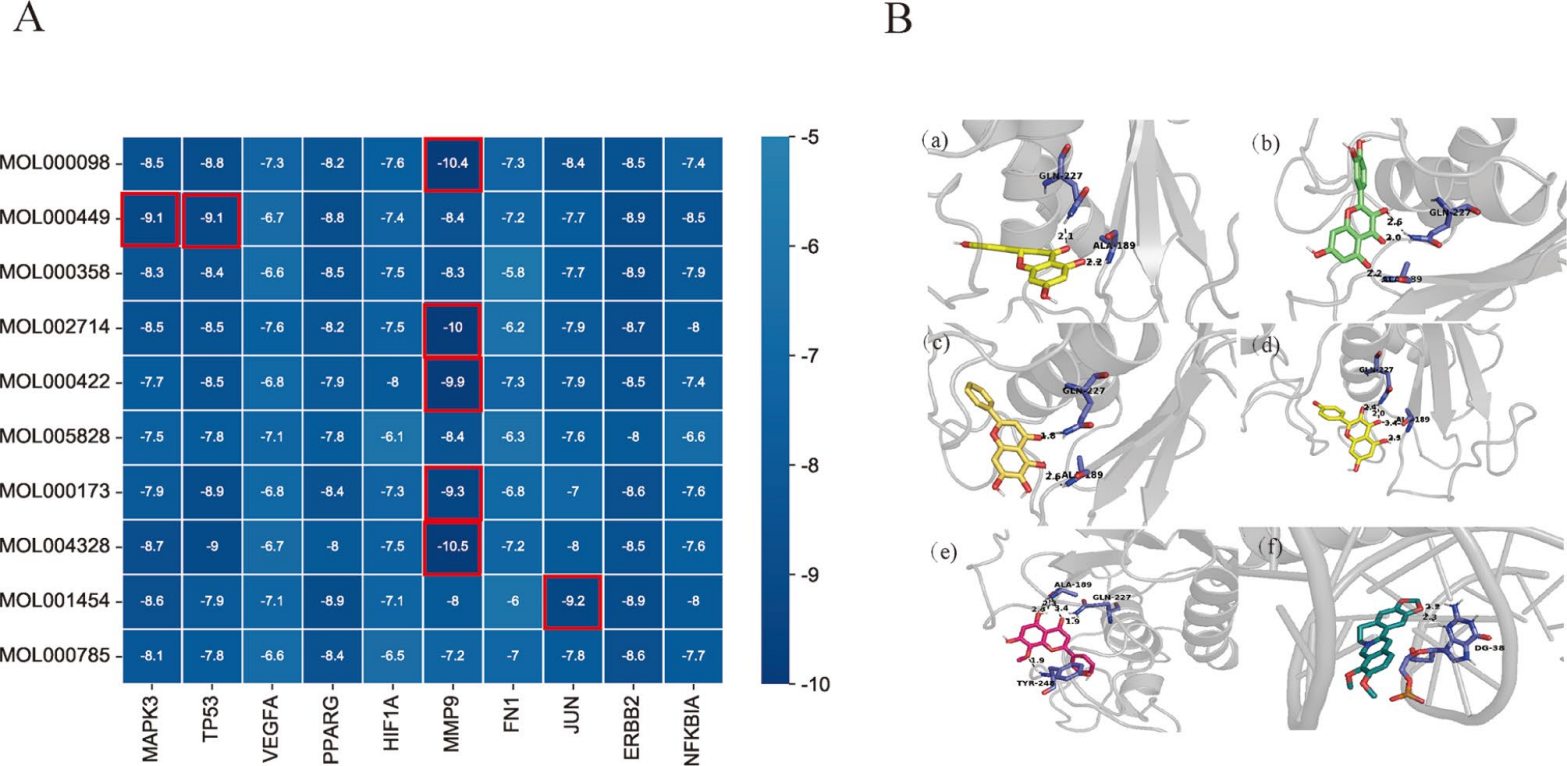
Validation of JCJ-PD target by molecular docking analysis. **(A)** Molecular docking heatmap of PD related targets with main compounds of JCJ. **(B)** Molecular docking patterns of the top 6 absolute values of the docking score: naringenin (MOL004328) binds to MMP-9 (a), quercetin (MOL000098) to MMP-9 (b), baicalein (MOL002714) to MMP-9 (c), kaempferol (MOL00422) to MMP9 (d), wogonin (MOL000173) to MMP9 (e), berberine (MOL001454) to JUN (f). The hydrogen bonds were indicated by dashed lines

We selected the top 10 core active components and 10 core targets for molecular docking. The core active components (quercetin, stigmasterol. beta-sitosterol, etc.) were conducted molecular docking with AutoDock Vina. The heatmap of absolute values of the docking score were shown in Fig. 6a. The molecular docking results showed that the binding ability of naringenin, quercetin, baicalein, kaempferol, wogonin to MMP9 was strong (MMP9-naringenin, affinity value=-10.5 kcal/mol; MMP9-quercetin, affinity value=-10.4 kcal/mol; MMP9-baicalein, affinity value=-10 kcal/mol; MMP9- kaempferol, affinity value=-9.9 kcal/mol; MMP9-wogonin, affinity value=-9.3 kcal/mol). In addition, the binding ability of berberine to JUN (JUN-berberine, affinity value=-9.2 kcal/mol), Stigmasterol to MAPK3 and TP53 were also strong (MAPK3-Stigmasterol, affinity value=-9.1 kcal/mol; TP53- Stigmasterol, affinity value=-9.1 kcal/mol). The 3D binding patterns of the target proteins and components were processed and visualized by PyMoL2.3.0 (Fig. 6b). The affinity value of the best binding postures was calculated (Table 4).

**Table 4:** Affinity value of the protein and compounds

ID	MolName	Target name (Affinity kcal/mol)										
		MAPK3	TP53	VEGFA	PPARG	HIF1A	MMP9	FN1	JUN	ERBB2	NFKBIA	
MOL000098	quercetin	-8.5	-8.8	-7.3	-8.2	-7.6	-10.4	-7.3	-8.4	-8.5	-7.4	
MOL000449	Stigmasterol	-9.1	-9.1	-6.7	-8.8	-7.4	-8.4	-7.2	-7.7	-8.9	-8.5	
MOL000358	beta-sitosterol	-8.3	-8.4	-6.6	-8.5	-7.5	-8.3	-5.8	-7.7	-8.9	-7.9	
MOL002714	baicalein	-8.5	-8.5	-7.6	-8.2	-7.5	-10	-6.2	-7.9	-8.7	-8	
MOL000422	kaempferol	-7.7	-8.5	-6.8	-7.9	-8	-9.9	-7.3	-7.9	-8.5	-7.4	
MOL005828	nobiletin	-7.5	-7.8	-7.1	-7.8	-6.1	-8.4	-6.3	-7.6	-8	-6.6	
MOL000173	wogonin	-7.9	-8.9	-6.8	-8.4	-7.3	-9.3	-6.8	-7	-8.6	-7.6	
MOL004328	naringenin	-8.7	-9	-6.7	-8	-7.5	-10.5	-7.2	-8	-8.5	-7.6	
MOL001454	berberine	-8.6	-7.9	-7.1	-8.9	-7.1	-8	-6	-9.2	-8.9	-8	
MOL000785	palmatine	-8.1	-7.8	-6.6	-8.4	-6.5	-7.2	-7	-7.8	-8.6	-7.7	

## Discussion

In our study, with transcriptome sequencing and a network pharmacology-based approach, a total number of 47 genes were identified as JCJ-PD-related targets. Further analysis was conducted within these targets with PPI analysis, C-D-T network analysis, and enrichment analysis. Results demonstrated that MAPK3, TP53, VEGFA, PPARG, HIF1A, MMP9, FN1, JUN, ERBB2, NFKBIA were considered as top 10 targets; quercetin, stigmasterol, beta-sitosterol, baicalein, kaempferol, nobiletin, wogonin, naringenin, berberine, palmatine were most important anti-PD bioactive compounds. Predicted JCJ-PD genes were mostly involved in oxidative stress, and chemical stress processes, and enriched mainly in HIF-1 and IL-17 signaling pathways. In addition, MMP9 was able to stably bound with several bioactive components such as naringenin, quercetin, and baicalein, which was validated by molecular docking. Our main findings not only identified the potential molecular mechanism of JCJ against PD but also paved the way for future therapeutic and mechanistic studies of novel agents for treating PD.

TCM a staple of medical practice for thousands of years within the Chinese community and is now increasingly recognized as an alternative and natural therapy for treating a variety of diseases [25–27]. Moreover, many purified chemical drugs used in clinical trials are extracted from herbal plants of TCM, e.g., artemisinin, a natural product of anti-malarial drug, was extracted from *Artemisia annua L*. by Youyou Tu’s team [28]. Nowadays, TCM has made remarkable achievements in the research and treatment of PD because of its advantages of multi-component, multi-target, and multi-stage treatment effects [29–31]. However, inferring the mechanisms of TCM, finding a novel candidate for treating PD remains challenging.

Transcriptome sequencing is a commonly used technique for identifying differential expression in case–control or cohort studies [32]. In our study, we enrolled 12 PD patients and 12 matched controls. On average, we obtained 6.86 GB clean data in each sample, and around 46 million clean reads were assembled after filtered and trimmed the raw data. With further analysis, 2669 DEGs between PD and control were identified, in which 1914 were down-regulated genes and 755 were up-regulated genes. Whether there is a regulatory association between JCJ and these genes needs to be further clarified.

JCJ is a classical herbal formula that has been administered for PD in clinical practice in China [9, 10]. JCJ consists of six herbal drugs, i.e., *Angelica sinensis*, *Achyranthes bidentata*, *Aurantii Fructus*, *Rhizoma Cimicifugae*, *Cistanche deserticola* and *Alisma orientalis*. As predicted by network pharmacological methods, a total number of 38 initial chemical components of JCJ and 260 targets were finally identified. Finally, were overlapping with the results of transcriptome sequencing, and considered as JCJ-PD-related genes.

Of these 47 genes, MAPK3, TP53, and VEGFA were recognized as the most crucial genes that participated in the JCJ treatment of PD. MAPK3 (also known as ERK1), an key member of Mitogen-activated protein kinase (MAPK) signaling pathway, its phosphorylation can be activated by a MAPK kinase (MAPKK) [33]. Previous studies have demonstrated that MAPK3 is required for mammalian normal neural crest development [34]. In PD rat model, stimulation of MAPK signaling is involved in mediating the neuroprotective effects of Nootkatone [35]. Others have found that inhibition of MAPK3 conferred protection against mutant LRRK2-induced neurite shortening in PD-induced cognitive dysfunction [36]. TP53 is commonly known as a tumor suppressor gene which more likely to mutate in the cancer development [37]. Moreover, it also involved in regulation of dopaminergic neuronal cell death or neuronal terminal damage [38]. A single-cell genomic profiling of human dopamine neurons showed that upregulation of the transcription factor encoded by TP53 provides a link to PD, in which TP53 has been implicated in motor neuron death [39]. Prior research has indicated that TP53-induced glycolysis and apoptosis regulator (TIGAR) plays a significant role in the oxidative stress-induced damage of dopamine neurons in PD induced by the MPTP [40]. Furthermore, studies have shown that vascular endothelial growth factor A (VEGFA) is predominantly expressed in astrocytes and contributes to the disruption of the blood-brain barrier in PD, as previously reported [41, 42].

Based on the GO and KEGG pathway analyzed results, JCJ may treat PD through biological processes such as response to oxidative stress, response chemical stress. In addition, most of its targets were enriched in transcription regulatory complexes and DNA binding transcription factor. As for signaling pathways, Hypoxia inducible factor-1 (HIF-1) signaling pathway, exhibiting the highest gene count enrichment among the PD-related signal pathways. HIF-1 is an oxygen tension dependent transcription factor, which is responsible for cell adaptation and survival under hypoxia [43]. HIF-1 regulates the expression of many target genes and participates in many cellular processes, e.g., substance synthesis, glucose energy metabolism, cell proliferation and cell phase regulation [44, 45]. Many evidences show that HIF-1 is related to the etiology, disease progression, treatment and other processes of PD [46].

This study showed that the core components of JCJ to improve PD were quercetin, stigmasterol, beta-sitosterol, baicalein, kaempferol, nobiletin, wogonin, naringenin, berberine, and palmatine. Combined with the results of molecular docking, it is revealed that naringenin, quercetin, baicalein, kaempferol and wogonin have the best binding performance with cytokines and matrix metalloproteinases-9 (MMP-9). MMP9 is a type IV collagenase and a member of the endopeptidase family of proteins, which highly expressed in the brain and recognized as a unique player in brain physiology and pathology [47, 48]. Previous studies have shown that naringenin could attenuated diabetic neuropathic pain by modulating the expression of MMP-9 [49], and may have protective effect on ischemic stroke by down-regulating the expression of NOD2, RIP2, NF-κB, MMP-9 [50]. In recent years, several studies have focused on quercetin’s effect on MMP-9, e.g., quercetin could inhibit the expression of MMP-9 via the AKT and ERK signalling pathways in gliomas [51]. In addition, Zhao et al.’s study demonstrated that quercetin improved the blood-brain barrier dysfunction by increasing the expression of ZO-1, Claudin-5, β-catenin, and LEF1, and decreasing the expression of MMP-9, GSK-3β and Axin in cerebral ischemia reperfusion model [52]. Baicalein also showed a protective effect on hippocampal neuronal damage by inhibiting MMP-9 activity [53]. At present, little is known regarding the effect of kaempferol and wogonin on MMP-9 in neurological diseases model. Our study might provide the new views for bioactive compounds of TCM in treating PD.

However, the present study has several limitations: (1) In this study, we employed transcriptome sequencing using peripheral blood samples obtained from patients diagnosed with PD. While the results obtained from this analysis may hold potential for the diagnosis of PD, it is important to note that diagnostic-related investigations have not yet been performed. (2) There is lack of pharmacodynamic and molecular biologic experiments. Additional animal studies or even clinical trials are desperately required to further verify our research results.

## Conclusion

To sum up, based on transcriptome sequencing, network pharmacology and molecular docking, this study preliminarily explored the potential mechanism of JCJ in the treatment of PD, which provides a new research direction for further exploring the mechanism of JCJ in the treatment of PD, and also provides a theoretical basis for the clinical application of JCJ in the treatment of PD. However, further pharmacological and animal experiments need to be carried out in the later stage to confirm the main regulatory target of JCJ for PD.

## Electronic supplementary material

Below is the link to the electronic supplementary material.

**Additional file 1**

## Data Availability

The raw sequence data reported in this paper have been deposited in the Genome Sequence Archive (Genomics, Proteomics & Bioinformatics 2021) in national Genomics data Center (Nucleic Acids Res 2022), China National Center for Bioinformation / Beijing Institute of Genomics, Chinese Academy of Science (GSA-Human: HRA004091) that are publicly accessible at https://ngdc.cncb.ac.cn/gsa-human/.

## Abbreviations

BP: Biological process
CC: Cellular component
C-D-T: Compound-Disease-Target
DEGs: Differentially expressed genes
DL: Drug likeness
GO: Gene Ontology
HIF-1α: Hypoxia inducible factor-α
JCJ: Ji Chuan Jian
KEGG: Kyoto Encyclopedia of Genes and Genomes
MAPK: Mitogen-activated protein kinase
MF: Molecular function
MMP9: Matrix metalloproteinases-9
OB: Oral bioavailability
PD: Parkinson’s disease
PPI: Protein-protein interaction
TCM: Traditional Chinese Medicine
TCMSP: Traditional Chinese Medicine System Pharmacology
