# Supplementary material for: Transcriptome sequencing and network pharmacology-based approach to reveal the effect and mechanism of Ji Chuan Jian against Parkinson’s disease
Source: BMC Complement Med Ther. 2023 Jun 3;23:182. doi: 10.1186/s12906-023-03999-6 (PMC10239169; doi:10.1186/s12906-023-03999-6)
Supplement: Supplementary file 1 — Additional file 1 [file 12906_2023_3999_MOESM1_ESM.docx]

Supplementary Material

**Supplemental Table 1 Summary of characteristics of PD patients and healthy controls**

| Category | Control (n=12) | PD (n=12) | *P* value |
| --- | --- | --- | --- |
| Age (Mean ± standard deviation) | 70.67±5.57 | 71.83±5.04 | 0.596 |
| Gender |  |  |  |
| Female (n, %) | 6, 50% | 6, 50% | 0.682 |
| Male (n, %) | 5, 42% | 7, 58% |  |
| BMI (kg/m^2^) | 23.85±2.26 | 24.25±2.09 | 0.659 |
| Disease duration | - | 4.83±1.9 | - |

**Supplemental Table 2 Summary of the transcriptome data**

| Sample | Raw reads | Clean reads | Clean bases | Q20 (%) | Q30 (%) | GC (%) |
| --- | --- | --- | --- | --- | --- | --- |
| Control 1 | 43170712 | 42369222 | 6.36G | 98.05 | 94.35 | 54.72 |
| Control 2 | 47854426 | 46914388 | 7.04G | 98.02 | 94.42 | 56.07 |
| Control 3 | 53369856 | 51323894 | 7.7G | 98.21 | 94.75 | 57.37 |
| Control 4 | 46920226 | 46078168 | 6.91G | 97.95 | 94.22 | 54.37 |
| Control 5 | 48061960 | 47110196 | 7.07G | 98.02 | 94.37 | 54.48 |
| Control 6 | 46018494 | 45108160 | 6.77G | 98.24 | 94.81 | 55.26 |
| Control 7 | 45299816 | 44219138 | 6.63G | 97.92 | 94.04 | 54.12 |
| Control 8 | 45825962 | 44492752 | 6.67G | 98.29 | 95.02 | 56.79 |
| Control 9 | 47817812 | 45931536 | 6.89G | 98.14 | 94.69 | 56.05 |
| Control 10 | 46958700 | 46135134 | 6.92G | 97.63 | 93.51 | 51.69 |
| Control 11 | 46618858 | 45659598 | 6.85G | 97.96 | 94.26 | 53.17 |
| Control 12 | 41277844 | 40573624 | 6.09G | 97.97 | 93.92 | 53.18 |
| PD 1 | 47466768 | 46279802 | 6.94G | 98.13 | 94.71 | 57.16 |
| PD 2 | 47894650 | 46838456 | 7.03G | 98.06 | 94.46 | 55.21 |
| PD 3 | 46744868 | 45844378 | 6.88G | 97.92 | 94.16 | 54.64 |
| PD 4 | 48006142 | 47063730 | 7.06G | 97.94 | 94.25 | 55.18 |
| PD 5 | 46767244 | 45945416 | 6.89G | 97.85 | 93.92 | 52.89 |
| PD 6 | 46081494 | 45309638 | 6.8G | 98.01 | 94.18 | 53.29 |
| PD 7 | 45670278 | 44537298 | 6.68G | 98 | 94.33 | 55.02 |
| PD 8 | 45255316 | 44468960 | 6.67G | 98.2 | 94.64 | 53.96 |
| PD 9 | 47147168 | 46082598 | 6.91G | 97.93 | 94.06 | 53.78 |
| PD 10 | 45749318 | 44632554 | 6.69G | 97.57 | 93.42 | 56.1 |
| PD 11 | 47616820 | 46665576 | 7.0G | 98.16 | 94.57 | 54.37 |
| PD 12 | 49656444 | 48564926 | 7.28G | 97.95 | 94.25 | 54.23 |

**Supplemental Table 3 Summary of the reads mapped to reference genome**

| Sample | Total reads | Total map | Unique map | Multi map |
| --- | --- | --- | --- | --- |
| Control 1 | 42369222 | 41083772(96.97%) | 33073037(78.06%) | 8010735(18.91%) |
| Control 2 | 46914388 | 45515377(97.02%) | 34577746(73.7%) | 10937631(23.31%) |
| Control 3 | 51323894 | 50211363(97.83%) | 33532207(65.33%) | 16679156(32.5%) |
| Control 4 | 46078168 | 44590824(96.77%) | 37875751(82.2%) | 6715073(14.57%) |
| Control 5 | 47110196 | 45751172(97.12%) | 36954191(78.44%) | 8796981(18.67%) |
| Control 6 | 45108160 | 43996115(97.53%) | 33189061(73.58%) | 10807054(23.96%) |
| Control 7 | 44219138 | 42869787(96.95%) | 34812944(78.73%) | 8056843(18.22%) |
| Control 8 | 44492752 | 43564311(97.91%) | 29724817(66.81%) | 13839494(31.11%) |
| Control 9 | 45931536 | 44715627(97.35%) | 31856188(69.36%) | 12859439(28.0%) |
| Control 10 | 46135134 | 44399599(96.24%) | 41829127(90.67%) | 2570472(5.57%) |
| Control 11 | 45659598 | 44318147(97.06%) | 38775347(84.92%) | 5542800(12.14%) |
| Control 12 | 40573624 | 39323716(96.92%) | 34009046(83.82%) | 5314670(13.1%) |
| PD 1 | 46279802 | 45092868(97.44%) | 30801506(66.55%) | 14291362(30.88%) |
| PD 2 | 46838456 | 45545650(97.24%) | 35324622(75.42%) | 10221028(21.82%) |
| PD 3 | 45844378 | 44362748(96.77%) | 36102581(78.75%) | 8260167(18.02%) |
| PD 4 | 47063730 | 45668293(97.04%) | 36113207(76.73%) | 9555086(20.3%) |
| PD 5 | 45945416 | 44483826(96.82%) | 38642211(84.1%) | 5841615(12.71%) |
| PD 6 | 45309638 | 44041227(97.2%) | 37335211(82.4%) | 6706016(14.8%) |
| PD 7 | 44537298 | 43322322(97.27%) | 33430175(75.06%) | 9892147(22.21%) |
| PD 8 | 44468960 | 43377152(97.54%) | 34827177(78.32%) | 8549975(19.23%) |
| PD 9 | 46082598 | 44594889(96.77%) | 36633939(79.5%) | 7960950(17.28%) |
| PD 10 | 44632554 | 43227707(96.85%) | 31090329(69.66%) | 12137378(27.19%) |
| PD 11 | 46665576 | 45439650(97.37%) | 37375107(80.09%) | 8064543(17.28%) |
| PD 12 | 48564926 | 47099624(96.98%) | 38440009(79.15%) | 8659615(17.83%) |

**Supplemental Table 4 Statistical analysis of the area of reference genome mapped with reads**

| Sample | exon | intron | intergenic |
| --- | --- | --- | --- |
| Control 1 | 5879365825(95.6572%) | 175707964(2.8588%) | 91218594(1.4841%) |
| Control 2 | 6562162852(96.3441%) | 163789757(2.4047%) | 85220087(1.2512%) |
| Control 3 | 7224686301(96.2233%) | 179816788(2.3949%) | 103750707(1.3818%) |
| Control 4 | 6389297613(95.7647%) | 186382687(2.7936%) | 96194636(1.4418%) |
| Control 5 | 6488019531(94.7998%) | 248073592(3.6247%) | 107819389(1.5754%) |
| Control 6 | 6098318375(92.6377%) | 384271706(5.8374%) | 100381597(1.5249%) |
| Control 7 | 5833631731(90.9693%) | 458853530(7.1553%) | 120261328(1.8753%) |
| Control 8 | 6277747318(96.4021%) | 169148270(2.5975%) | 65145275(1.0004%) |
| Control 9 | 6319943417(94.5404%) | 273284989(4.0881%) | 91685886(1.3715%) |
| Control 10 | 5769561148(86.8253%) | 673555093(10.1362%) | 201902365(3.0384%) |
| Control 11 | 5983116566(90.1878%) | 501712058(7.5627%) | 149242139(2.2496%) |
| Control 12 | 5397631932(91.7122%) | 373676497(6.3492%) | 114096604(1.9386%) |
| PD 1 | 6558733814(97.2375%) | 119353342(1.7695%) | 66980346(0.993%) |
| PD 2 | 6462655943(94.819%) | 267460542(3.9241%) | 85667208(1.2569%) |
| PD 3 | 6282306784(94.6409%) | 255729043(3.8525%) | 100016351(1.5067%) |
| PD 4 | 6601419231(96.595%) | 117225752(1.7153%) | 115476923(1.6897%) |
| PD 5 | 5825306658(87.5273%) | 670492066(10.0744%) | 159618475(2.3983%) |
| PD 6 | 5828456838(88.4254%) | 616680901(9.3559%) | 146248797(2.2188%) |
| PD 7 | 6120362614(94.3882%) | 266274761(4.1065%) | 97608999(1.5053%) |
| PD 8 | 6039555767(93.0567%) | 350979259(5.4078%) | 99654477(1.5355%) |
| PD 9 | 5880610807(88.1546%) | 655980476(9.8336%) | 134206707(2.0119%) |
| PD 10 | 6260612978(96.8292%) | 131898852(2.04%) | 73106850(1.1307%) |
| PD 11 | 6498341850(95.5645%) | 209846653(3.086%) | 91768202(1.3495%) |
| PD 12 | 6298540023(89.3842%) | 620682281(8.8083%) | 127371224(1.8076%) |
